# When to decide to enroll a TTR-FAP patient in a Clinical Trial?

**DOI:** 10.1186/1750-1172-10-S1-I4

**Published:** 2015-11-02

**Authors:** Juan Buades

**Affiliations:** 1 Hospital Clínico San Carlos, Madrid, Spain

## Background

The Transthyretin-Familial Amyloid Polyneuropathy (TTR-FAP) is a disease caused by deposition of mutant transthyretin (TTR), produced approximately 95% in the liver and the rest in the plexus choroideus and retina. In 1990 the first TTR-FAP patient liver transplant was performed. The liver transplant, which suppresses TTR synthesis, was the only treatment available to modify this disease until 2011, when the European Medicines Agency approved Tafamidis, a TTR stabilizer, for stage I patients (Val30Met and non-Val30Met). To improve upon the pre-existing therapy of liver transplants and given the results of 68% of Tafamidis respondents, other disease-modifying therapeutic approaches were developed: Silencers, Stabilizers, Degraders and Reabsorption agents. In light of these multiple agents clinical trials are the key to improve TTR-FAP treatment.

## Objectives

The aim of the analysis is to determine eligibility criteria and optimal timing of patient’s enrollment in a clinical trial.

## Methods

We reviewed the literature using the online database PUBMED. Standard search strategies were applied using the following MESH terms alone or in combination: amyloidosis, transthyretin, liver transplant, tafamidis and clinical trial. Evaluation of the bibliographies was done in order to select relevant articles. Additionally, clinical experience achieved by lead investigators and our own experience was applied in this analysis.

## Results

Nowadays siRNA (Patisaran®), Antisense Oligonucleotides, Diflunisal and Doxycicline-TUDCA are under clinical trial investigation. Election criteria definition is essential to enroll a patient in one of those clinical trials. Based on the bibliography, inclusion criteria protocols of each clinical trial and our own experience the following criteria have been developed:

1. Stage I to Stage IIIB can be recruited into a clinical trial at any time.

2. Stage I non-respondents to Tafamidis after 12 months treatment who show progression of the disease indicated by

• Worsening of ambulation (increase of PND score by one point)

• Onset of orthostatic hypotension or impotence

• Progress of cardiomyopathy with worsening of stage of cardiac insufficiency NYHA by 1 point or of cardiac conduction disorders

3. It is expected than in the short future OLT patients who underwent illness progression would be enrolled in a clinical trial.

## Conclusions

Disease staging, tafamidis response and OLT limitations are the main factors to be considered before recruiting a patient for a clinical trial.

